# Supporting autistic communities through parent-led and child/young person-led digital social story interventions: an exploratory study

**DOI:** 10.3389/fdgth.2024.1355795

**Published:** 2024-03-13

**Authors:** Louis John Camilleri, Katie Maras, Mark Brosnan

**Affiliations:** Centre for Applied Autism Research (CAAR), University of Bath, Bath, United Kingdom

**Keywords:** autism, children and young people (CYP), parents, social stories, digital mediation, digital intervention

## Abstract

**Introduction:**

Social Stories (SS) is a socially-valid intervention for autistic children and young people (CYP) which is used widely by professionals and parents. Research suggests that whilst parents are in an ideal position to deliver interventions for their autistic CYP, a lack of procedural integrity can result in a great deal of variability in parent-mediated intervention outcomes.

**Methods:**

This exploratory study investigated the extent to which SS can be effectively developed and delivered, through digital mediation, by parents with little to no researcher input (*n* = 17, sample 1) and the factors that impact effectiveness. Furthermore, the study also investigated the extent to which digitally-mediated SS can support autistic CYP to develop and deliver their own stories, thereby utilising the intervention as a means for self-support and self-management (*n* = 5, sample 2).

**Results:**

The outcomes of the study indicate that digital mediation can effectively support parent-led SS intervention. Findings also indicate that receptive/expressive language skills of autistic CYP, their level of systemizing, as well as the practice of consulting with the autistic CYP whilst identifying goals and developing stories, are individual and procedural characteristics which positively influence the effectiveness of the parent-led intervention. The study also found that digitally-mediated SS can be utilised as a self-support tool by autistic CYP themselves.

**Discussion:**

The results inform the developing literature on digital interventions and support tools that aim to engage with, and involve further, the autistic community in the setting and authoring of interventions and research.

## Introduction

Research focused on supporting autism has increasingly highlighted the need for there to be greater involvement and participation of the autistic and broader autism (e.g., parents) communities both in the setting and authoring of interventions and research (1–3). As a result, there has been an increasing interest in parent-led interventions (4) as opposed to clinician-led interventions (i.e., interventions that are delivered by trained professionals, such as therapists, psychologists, or behaviour analysts), as a means to engage the autistic and broader autism communities. Parents (and/or guardians, caregivers, and advocates—hereafter referred to as parents) are in an ideal position to deliver and support their children's or young person's (CYP) interventions (5, 6), especially when considering the intimate insights, knowledge and experiences of and on their CYP and their profiles. There is growing evidence of an increase in the involvement of parents of autistic CYP in implementing support practices and interventions and being more frequently involved in attempts to support their CYP (7–9).

While Parent-Mediated Interventions (PMI) have the potential to positively influence the lives of autistic CYP and their families (10), variability has been reported in terms of outcomes (8, 11). Reasons for this variability are numerous (10), and include treatment fidelity [i.e., the extent to which an intervention is implemented as intended—see (12)] as well as ill-defined parameters of PMI. Bearss et al. (13) attempt to reduce the ambiguity surrounding the definition by defining PMI as an activity that actively engages the parent in promoting skill acquisition or behaviour change in the CYP. PMIs are technique-focused interventions where the parent is the agent of change and the CYP is the direct beneficiary. Furthermore, Bearss et al. also differentiate between primary and complimentary programs. Complimentary programmes place parents in the context of a therapist-led intervention team, whilst primary programs have the parents actively engaged from the outset in order to facilitate the CYP's acquisition of specific skills.

One example of a technique-focused primary intervention is Social Stories^TM^ (SS). SS is a popular intervention that is frequently utilised by parents to assist autistic CYP (14). SS is regarded as a useful and effective technique to support autistic CYP in their primary socialisation contexts and with individuals close to them (15, 16). The SS intervention was introduced by Gray and Garand (17) and consists of structured scripts comprising individualised text and illustrations which aim to facilitate the transfer of information between the author (typically a professional or a parent) of the story and the audience (the CYP). To be classified as a SS, stories have to meet ten criteria (18–20), which include having one goal for each story, having a story which is more descriptive than it is directive, and having a story that answers “where”, “when”, “who”, “what”, “how”, and “why” questions. The SS are developed, usually by the authors, in a positive and guiding tone/voice, and are then read to the CYP or read by the CYP themselves. SS is one of the most frequently used interventions to support autistic CYP (21), and is consistently positively rated by parents of autistic CYP (22). Notwithstanding its popularity, SS outcome research reports a high degree of variability in terms of effectiveness: Systematic meta-analyses of literature describe SS as highly effective (14, 23), moderately effective (24) and even as questionable (25). Treatment fidelity could be one of the factors influencing such variability (15). Furthermore, it is reported that the effectiveness of SS can also be influenced by child characteristics such as language and communication skills (26, 27), and also by the type of goals which SS target. Kokina and Kern (25) analysis of the SS literature highlights that SS has been employed frequently to support autistic CYP in four main goal areas: (1) the reduction of inappropriate behaviours, (2) the development of social skills, (3) the teaching of academic skills, and (4) assistance with novel events/transitions.

Reviews of the SS literature have highlighted that little is known regarding the underlying psychological mechanisms related to SS effectiveness (15, 28). Camilleri et al. (29) suggest that the explicit structure of SS may make uncertain situations more predictable for autistic CYP whilst building upon relative autistic strengths in “systemizing”. Systemizing is the drive to identify lawful regularities (often causal) that govern the input-operation-output relationships (30). SS can focus on relevant details of a situation (the input), describe a manipulation of the situation (the operation), and then explain the outcome (the output). From this perspective, SS can be seen as a strength-based approach to supporting autism (29).

The degree to which parents can independently develop and deliver the SS intervention effectively to their CYP is unclear. Furthermore, the literature on the effectiveness of the SS intervention, when delivered by parents, is sparse. A study by Ghanouni et al. (31) engaged parents and clinicians in a Delphi method procedure to develop and validate a library of 75 SS for autistic CYP. However, in Ghanouni et al.'s study the SS were not delivered. A study by Hutchins and Prelock (32) involved parents in the development of SS for autistic CYP, but not in the delivery of the SS. The outcomes of this study yielded positive socially valid outcomes in terms of addressing behavioural and communicative functioning of autistic CYP. However, Hutchins and Prelock's (32) study was researcher-led; that is, parents were mostly involved in the evaluation of outcomes rather than in the delivery of the intervention. Dodd (33) and Hutchins (32) also involved parents in the identification of goals and in the development of the SS. However, in both of these studies, the intervention was delivered by the researchers. Studies by Acar et al. (34, 35) involved parents and family members in the identification of goals for their autistic CYP, as well as in the development and delivery of their SS. Acar et al. (34) showed that SS intervention could be successful with parent-child (aged 6–10 years) dyads and Olçay-Gül (35) showed that SS intervention could be successful for parent-young person (aged 12–16 years) dyads. Outcomes from both studies indicate that SS intervention is effective in supporting social understanding for autistic CYP. Both studies involved face-to-face training for parents, indicating that parents can be effectively coached on the creation and implementation of SS [see also (36)].

While parents can deliver effective SS to autistic CYP with effective coaching, many parents do not receive coaching and there is huge variability in the effectiveness of parent-delivered SS [see (37)]. One potential method for providing coaching for the development and delivery of effective SS to autistic CYP has been the development of digitally-mediated SS. For example, Hanrahan et al. (38, 39) utilised a free-to-use digital application which was co-designed and co-developed with the autistic and broader autism communities [SOFA-app.org (40)] to test the effectiveness of digitally-mediated SS in producing beneficial changes in behavioural outcomes. However, this was not a PMI; although it resulted in a decrease in inappropriate behaviours (38), reduced perceived anxiety levels, and increased understanding in autistic CYP (39), in both studies the SS were developed by the researchers. A study by Smith, Toms, et al. (41) again used the SOFA-app to support 17 school teachers to develop and deliver SS to autistic CYP. The school teachers were provided with one brief training session on how to use the SOFA-app, which contains tutorials on Gray's criteria. The school teachers subsequently identified goals, and developed and delivered personalised digitally-mediated SS with 22 autistic children aged 5–11 years. The study indicated that digitally-mediated SS are effective at addressing behaviour, reducing anxiety and increasing understanding in autistic children. The outcomes of this study also indicated that digital mediation can support the delivery of the SS intervention with a high degree of treatment fidelity within a real-world school setting.

Thus, there is evidence that researchers and school teachers can develop and deliver effective digitally-mediated SS with a high degree of procedural integrity (i.e., reliable and accurate implementation of an intervention). In addition, recent research indicates that digitally-mediated SS also has the potential to support parents of autistic CYP in SS-writing competence whilst also improving intervention integrity (37, 42). However, it is unknown if digitally-mediated SS which have been developed and delivered by parents in a real-world setting can lead to similar outcomes as those reported for researchers and school teachers. The use of digitally-mediated technologies to support the broader autism community (such as parents, etc.) has been increasing in recent years (43). This increase may be as a result of the potential utility of technology within a real-world setting, by providing decreased social demands and higher predictable responses (44). Digital technology can also support the family as well as the autistic individual (45) and can be administered and utilised by the parent or by the autistic individual (46). Furthermore, technology can be used for self-support and self-management for autistic individuals (47). Similar to PMIs, self-management interventions (SMI) aim to upskill autistic individuals to enable the self-regulation of behaviour and emotions in a way that encourages independence and enhances the capacity to manage challenging situations (48). SMI research is another example of participatory design which emphasizes an individual's ability to recognize and manage their own emotions, behaviour, and goals. SMI is an alternative to interventions controlled by other agents or actors and allows autistic individuals to be more independent whilst decreasing prompt-dependency (49). Digital technologies can be invaluable in supporting self-set intervention outcomes for autistic individuals (46).

Involving parents of autistic CYP and autistic CYP themselves in the development and delivery of SS is consistent with a high level of community participation, characterised as “doing it together”, “getting the help we asked for” and “doing it ourselves” (50). Through a two-sample pre-post design, this is the first study to investigate the effectiveness of digitally-mediated SS that are: (1) developed and delivered by parents of autistic CYP, and (2) self-developed and self-delivered by autistic CYP. The study also sought to explore the target goals of the SS intervention identified by parents and autistic CYP in addition to influential factors related to Gray's criteria, such as whether the parent involved the autistic CYP in the development of the SS. This study also sought to investigate if CYP's language skills (receptive and expressive) and systemizing drive (which is considered an autistic strength) can influence SS outcomes.

## Methods

### Recruitment

#### Sample 1 recruitment (parents)

Digitally-mediated SS studies have reported a medium to large effect size when using a pre-post design (39, 41). In line with these findings, an *a priori* power analysis using G*Power version 3.1.9.7 (51) indicated that for this study to detect a medium to large effect size of 0.8, a minimum sample size of 15 parents was necessary. The final sample consisted of 17 parents of autistic children. The parents who participated were required to be 18 years or over, and with children aged 4–16 years of age who had a clinical diagnosis of ASD assigned by a clinician using DSM-IV or DSM-V criteria. Parents also needed access to a digital device (such as a smartphone or tablet). Parents responded to an online advertisement to take part in parent-mediated social stories research. Participants from sample 1 received an appropriate honorarium as compensation for the time they dedicated to this project.

#### Sample 2 recruitment (autistic CYP)

A social stories SMI study for autistic adults (52) reported a large effect size. In line with these findings, an *a priori* power analysis using G*Power version 3.1.9.7 (51) indicated that a sample size of 5 was necessary to achieve Cohen's (53) recommended power of 0.8. The CYP who participated (*n* = 5) were required to be between 4 and 17 years of age with a clinical diagnosis of ASD assigned by a clinician using DSM-IV or DSM-V criteria. Participants also needed access to a digital device (such as a smartphone or tablet) and literacy skills commensurate with their age (or above). Participants were 2 children (aged 7 and 9) and 3 adolescents (aged 14, 15, and 16), who were all male. Sample 2 participants were distinct from Sample 1 participants (i.e., the CYP in sample 2 were not the same as the CYP in sample 1). Sample 2 participants were recruited through advertisements at a charity that supports parents of autistic CYP in Malta. The ASD diagnosis for Sample 2 was confirmed for the present study using the Autism Diagnostic Observation Schedule [ADOS-2 (54)] by the first author. The parents of Sample 2 received an honorarium towards expenses (such as travel for their CYP to undertake the ADOS).

### Stories online for autism (SOFA-app)

The stories used in this study were developed and delivered by the parents (Sample 1) and by the autistic CYP (Sample 2) with little to no support from the researchers through the use of the SOFA-app. The SOFA-app (sofa-app.org) is a free digital tool, for smartphone and tablet devices, which can be used on Android and iOS platforms. The SOFA-app was co-developed using a paradigm for participatory autism research that was aimed towards co-creating digital technologies together with the autistic and broader autism communities (2, 40, 55).

### Procedure and measures

#### Sample 1 procedure

**Figure 1:**
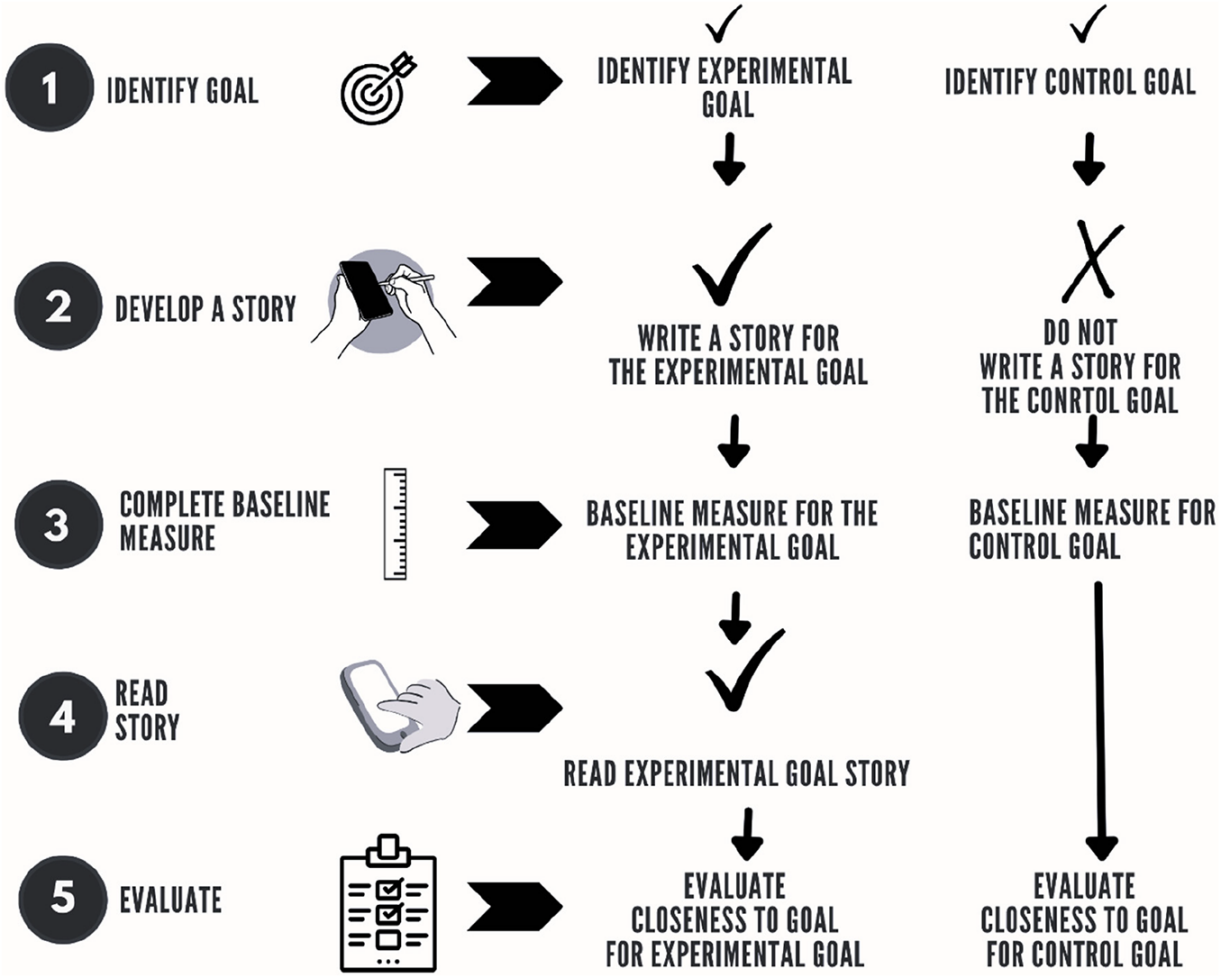
Procedure overview.

There was an initial online meeting between the researcher and each parent, where consent was obtained, the research was outlined, and any questions were answered. Parents were first asked to identify two intervention goals for their autistic CYP: an experimental goal and a control goal. A Social Story was developed for the experimental goal, whilst no story was developed for the control goal, which served as a point of comparison. Parents were encouraged to consult their autistic CYP and involve them as much as possible for the development of their SS. Parents were asked to download the SOFA-app and to deliver the SS through their digital devices, with their autistic CYP at least once every day for 2 weeks (38, 41, 56). The procedure is summarised in Figure 1. The design, therefore, matched previous research but extended from researchers and teachers developing and delivering the SS to parents developing and delivering the SS. The SOFA-app contains tutorials to support users in developing and delivering SS in a manner that conforms to Gray's guidelines. Parents were advised that additional tutorials were available via a YouTube channel, on how to download and use the SOFA-app^1^.

#### Sample 1 measures

The parents rated how close their children were to achieving their experimental and control goals, on an 11-point scale from 0 (Goal not at all reached) to 10 (Goal totally reached) at baseline (i.e., before starting to deliver the stories) and after 2 weeks of using the SOFA-app to deliver the stories with their children. The closeness-to-goal measure has been found to be the most reliable index of intervention effectiveness for SS (38, 41, 56, 57).

The Autism Quotient, AQ-10 (58), was completed by the parents as a check that the autistic CYP had high levels of autistic traits on this widely used screen for autism. The appropriate AQ-10 measure was completed for children (4–11 years) and for adolescents (12–16 years). The AQ-10 has a sensitivity of 0.93 and a specificity of 0.95 at a cut-point of 6. Parents also completed an age-appropriate systemizing quotient questionnaire for their autistic CYP. The 28 systemizing items for the SQ-Child version (59) had a high internal consistency (*α* = 0.78) and high intra-class correlation (*r* = 0.86). The 55 item SQ-Adolescent version (60) also had high internal consistency (*α* = 0.89) and test-retest reliability (*r* = 0.94).

Information about the parents' demographic and relevant experience, as well as their CYP's demographics are reported in Tables 1, 2 respectively. Parents also reported the perceived expressive (i.e., little to no understanding, single words or symbols, short sentences or conversations) and receptive (i.e., non or minimally verbal, uses single words, uses short sentences, uses full sentences) skills of their CYP. Parents in Sample 1 were all asked to consult with their children about the story goals and, at the end of the study, all the parents reported if they had consulted or if they had not consulted with their CYP. Parents also self-reported their own level of English literacy skills. An 11-point scale (0 to 10) was used to self-evaluate the parents' perceived English writing, reading and comprehension skills [see (39, 41, 56)].

**Table 1:** Sample 1 parent characteristics.

	Frequency (*n*)	Percentage (%)
Total number of parents/guardians	17	100
Gender		
Female	15	88
Male	2	12
Nationality		
Brazilian	1	6
British	8	47
Indian	1	6
Maltese	7	41
Race/ethnicity		
Asian/British Asian	2	12
Hispanic/Latino	1	6
White	14	82
Highest educational qualification achieved		
Post-secondary education completed	9	53
Tertiary education and beyond completed	8	47
Have you ever received training on how to develop, and deliver SS?		
Yes	4	76
No	13	24
How confident are you when using your smartphone or tablet to use android/iOS applications?		
Very confident	9	41
Confident	7	53
Neither confident nor unconfident	1	6
	Mean (SD)	Range
Age	40.06 (5.03)	31–49
Parental self-reported literacy skills		
Writing	9.00 (1.12)	7–10
Reading	9.29 (1.16)	7–10
Comprehension	9.12 (1.32)	6–10

SD, standard deviation; SS, social stories.

**Table 2:** Sample 1 CYP characteristics.

	Frequency (*n*)	Percentage (%)
Total number of children	17	100
Gender		
Female	4	23.5
Male	13	76.5
Nationality		
Brazilian	1	6
British	8	47
Indian	1	6
Maltese	7	41
Race/ethnicity		
Asian/British Asian	2	12
Hispanic/Latino	1	6
White	14	82
Diagnosis		
Autism spectrum disorder	17	100
Co-occurring		
Attention-deficit/hyperactivity disorder (ADHD)	1	6
Sensory processing disorder (SLP)	1	6
Tourette syndrome	1	6
Specific learning disability	2	12
Anxiety disorder	1	6
CYP's language skills (expressive & receptive)		
Full sentences & conversation	9	53
Short sentences/single words & simplified short sentences	8	47
	Mean (SD)	Range
CYP's age	8.18 (3.40)	4–15

At the end of the study the parents completed 12 evaluation questions which measures intervention appropriateness (61) and intervention feasibility (62) and have been used in similar research (52).

#### Sample 1 participant characteristics

The parents from Sample 1 were mostly white females who were educated to post-secondary level or above and confident with technology, see Table 1.

The parental descriptions of the autistic CYP are in Table 2. Parental ratings of their autistic CYP on the AQ10 screen was consistent with the diagnostic status (*M* = 7.76, SD = 1.52; range = 6–10). Mean parental ratings for their autistic CYPs systemizing was 25.59 (SD = 9.43, range = 12–46). The CYP were mostly white males, with relatively able expressive and receptive language. The mean age of the CYP was 8.18 years (SD = 3.40; range = 4 to 15).

#### Sample 2 procedure

Parents were asked to introduce the study to their autistic CYP and invite them to participate in the study. When discussing the protocol for the CYP, parents raised the issue that a 1-week intervention would be more viable for a SMI than the usual 2 weeks for PMI. As the 2-week protocol has demonstrated significant effects for autistic CYP after the first week when administered by researchers and school teachers (38, 39, 41, 56), a 1-week protocol was finalised. Parents completed the SRS-2 (63) and AQ10 (58) as a screen for autism. If the CYP were above cut off on these measures and agreed to participate, they were invited to complete an assent form (all those approached were above cut off and did assent). The CYP then met with the researcher (first author) in person and an ADOS (54) was administered. The Researcher and CYP then discussed how to use the SOFA-app to create a SS. Subsequently, the autistic CYP identified a goal for themselves, and developed a SS that aimed to support themselves in reaching that goal. The CYP was asked to complete a baseline closeness-to-goal measure and was subsequently invited to access and read the SS independently, using the SOFA-app, every day for 1 week. CYP were told that if they had any questions, they could ask their parent (the parent had access to the Researcher, but this was not needed). The CYP completed an online progress checklist at the end of the week as well as an in-person meeting was held which involved an evaluation of their experience of the study.

#### Sample 2 measures

The CYP completed a closeness-to-goal measure on an 11-point scale from 0 (Goal not at all reached) to 10 (Goal totally reached), at baseline (i.e., before starting to deliver the stories) and after 1 week of using the SOFA-app to access and read the SS. In addition, parents were invited to act as “independent raters”. Parents were asked to take into consideration the baseline rating identified by their child/adolescent and subsequently rate their perception of their CYP's progress towards reaching the goal in terms of a closeness-to-goal outcome rating identical to that used by their CYP. Finally, evaluative questions aimed towards measuring various aspects of the CYP's experience of the study were administered online.

#### Sample 2 participant characteristics

For Sample 2, an ADOS was administered to each CYP to confirm their ASD diagnosis. In addition, parents completed the SRS-2 and AQ10 for their autistic CYP. Parents also rated their CYP's literacy abilities (i.e., reading, comprehension, writing, and spelling skills) and confidence in using a smartphone, see Table 3. All CYP met criteria for an ASD diagnosis, had relatively high literacy skills and were confident in using smartphones. The AQ10 scores of the CYP did not significantly differ between Sample 1 and Sample 2, *t*(20) = .280, *p* = .561.

**Table 3:** Sample 2 CYP autism diagnosis scores and literacy scores.

Variables	Mean (SD)	Min—Max
CYP age	12.2 (3.66)	7–16
ADOS-2 overall total	9 (1.58)	7–11
ADOS-2 social affect score	6.2 (1.30)	5–8
ADOS-2 RRB score	2.8 (1.48)	1–5
SRS-2	75 (7.65)	63–84
AQ-10	8.2 (1.10)	7–10
English reading skills (0–10)	8.2 (1.30)	7–10
English comprehension skills (0–10)	7.4 (1.82)	5–10
English writing skills (0–10)	7.2 (1.64)	6–9
English spelling skills (0–10)	7.0 (1.87)	5–9
Confidence in using smartphone (1–5)	4.40 (0.55)	4–5

SD, standard deviation; Min, minimum; Max, maximum.

### Analysis

For both samples, paired-sample *t*-tests were used to compare closeness-to-goal ratings from baseline to outcome (i.e., after 2 weeks for experimental and control goals for Sample 1, and after 1 week for experimental goal for Sample 2). Paired-sample *t*-tests were used to analyse the difference in change in closeness-to-goal rating between experimental and control groups for Sample 1. An independent-samples *t*-test was run to determine if there were differences in change in closeness-to-goal rating between the parents who consulted and those who did not consult with their CYP and to determine if there were differences in closeness-to-goal ratings between children's receptive/expressive language. Effect size is reported as Cohen's d. A linear regression was run to understand the effect of systematizing quotients on change in closeness-to-goal ratings.

The goals that were identified by the parents in Sample 1, and by the CYP in Sample 2, were analysed in terms of Kokina and Kerns' (25) categories. The SS from Sample 1 of the study were also analysed in terms of Smith et al.'s (2020) framework for the evaluation of SS treatment fidelity. Sample 2 of the study was also analysed in terms of intervention characteristics (i.e., if the story was read daily, if reminders were required from an adult, and if the CYP required support).

### Ethics

This study received ethical approval from the University of Bath's Psychology Research Ethics Committee (PREC, 19-309) on January 16, 2020. Informed consent and/or assent was obtained from all participants.

## Results

### Change in pre and post closeness-to-goal for sample 1′s experimental goal

Pre and Post intervention measures were compared for Sample 1. Mean baseline closeness-to-goal was 3.12 (SD = 1.90) and outcome closeness-to-goal rating after 2 weeks was 7.41 (SD = 2.09). There was a statistically significant mean increase of 4.29, 95% CI [2.901, 5.688], *t*(16) = 6.533, *p* < .001. A Cohen's *d* of 1.58 indicates a large effect size, see Table 4.

**Table 4:** Overview of baseline and outcome ratings from samples 1 to 2.

	Mean	SD	Min	Max
Sample 1				
Experimental goal—baseline closeness-to-goal rating	3.12	1.90	0	7
Experimental goal—outcome closeness-to-goal rating	7.41	2.09	1	9
Experimental goal—change in closeness-to-goal rating	4.29	2.71	0	9
Control goal—baseline closeness-to-goal rating	2.82	2.82	0	7
Control goal—outcome closeness-to-goal rating	2.88	2.88	0	8
Control goal—change in closeness-to-goal rating	0.06	0.97	−3	1
Sample 2				
Child/adolescent's baseline closeness-to-goal rating	6.40	1.51	4	8
Child/adolescent's outcome closeness-to-goal rating	8.00	2.00	5	10
Child/adolescent's change in closeness-to-goal rating	1.60	1.14	0	3
Parent's outcome closeness-to-goal rating	7.40	2.20	4	10

SD, standard deviation; Min, minimum rating; Max, maximum rating.

### Change in pre and post closeness-to-goal for sample 1′s control goal

A control goal was selected at the beginning of the 2 weeks. The mean rating at baseline was 2.82 (SD = 1.94) and the mean outcome closeness-to-goal ratings after 2 weeks was 2.88 (SD = 2.18). The mean difference of 0.06 was not statistically significant, 95% CI [−0.353, 0.471], *t*(16) = 0.251, *p* = .805, Cohen's *d *= 0.06 see Table 4.

### Change in closeness-to-goal ratings between groups of CYP from sample 1 with different language skills

The parents were asked to rate their children's (Sample 1) receptive and expressive language skills: nine parents indicated that their child could understand conversation (receptive) and use full sentences to verbally communicate (expressive), whilst eight parents indicated that their child could understand simplified short sentences and use short sentences or single words to communicate verbally. The change in closeness-to-goal ratings were higher for CYP who understood conversation/used full sentences (*M* = 5.56, SD = 2.19) than for children who understood simplified sentences/used simplified sentence or single words (*M* = 2.88, SD = 2.64). The difference (*M* = 2.68) was statistically significant, 95% CI [0.19, 5.18], *t*(15) = 2.29, *p* = .037, Cohen's *d* = 1.17.

### Change in closeness-to-goal ratings between groups from sample 1 whose parents consulted/did not consult with the CYP

From the 17 participants in Sample 1, 8 consulted with their CYP during the development of the SS, and 9 did not. The change in closeness to goal was higher for parents who consulted with their CYP (*M* = 5.88, *SD* = 2.23) than parents who did not consult with their CYP (*M* = 2.89, *SD* = 2.37). The difference, *M* = 2.98, was statistically significant, 95% CI [0.598, 5.374], *t*(15) = 2.67, *p* = .018. Cohen's *d* = 1.30, see Table 5.

**Table 5:** Evaluation of SS treatment fidelity.

	Sample 1		Sample 2	
	Frequency	Percentage	Frequency	Percentage
Consulted CYP to develop goal and story				
Yes	8	47	Not applicable	
No	9	53		
Conform to Gray's criteria				
Criteria 1: the SS goal	17/17	100	5/5	100
Criteria 2: two step discovery^a^	8/17	47	5/5	100
Criteria 3: three parts and a title	12/17	70	3/5	60
Criteria 4: “FOURmat”^a^	17/17	100	5/5	100
Criteria 5: five factors define voice & vocabulary	15/17	88	5/5	100
Criteria 6: six questions that guide story development	17/17	100	5/5	100
Criteria 7: seven types of sentences	16/17	94	5/5	100
Criteria 8: A GR-EIGHT formula (sentence ratio)	9/17	53	1/5	20
Criteria 9: nine makes it mine^a^	8/17	47	5/5	100
Criteria 10: ten guides to editing & implementation	17/17	100	5/5	100

^a^ Criteria automatically met by self-developing SS in Sample 2.

### The impact of systemizing score on change in closeness-to-goal ratings (sample 1)

The effect of systematizing score on change in closeness-to-goal ratings was analysed. The Shapiro-Wilk *p* value (0.933) exceeded the.05 level of significance, indicating that the independent variable (systemizing score) distribution was normal. Systemizing scores significantly predicted change in closeness-to-goal scores, *F*(1, 15) = 15.81, *p* = .001, accounting for 48.1% of the variation in change in closeness-to-goal (adjusted *R*^2^). The prediction equation was: Change in closeness-to-goal = −1.59 + 0.21*systemizing score.

### Change in pre and post closeness-to-goal for experimental goal (sample 2)

Pre and Post intervention measures were compared for Sample 2. The mean closeness-to-goal rating at baseline was 6.40 (SD = 1.52) and outcome closeness-to-goal rating after 1 week of using the SOFA-app was 8.00 (SD = 2.00). There was a statistically significant mean increase of 1.60, 95% CI [0.184, 3.016], *t*(4) = 3.14, *p *= .035, *d* = 1.40.

### Change in pre and post closeness-to-goal for experimental goal (sample 2) according to their parents

For comparison purposes, parents also rated what they believed their CYP's closeness-to-goal rating to be after 1 week of reading the SS. The mean outcome ratings of parents (*M* = 7.40, SD = 2.20) was lower than the CYP outcome ratings (*M* = 8.00, SD = 2.00) with a difference of 0.6 (SD = 1.14). However, this difference was not statistically significant, 95% CI [−0.82, 2.02], *t*(4) = 1.18, *p* = .305, *d *= 0.53 (Table 4).

### Analysis of stories

The SS that were developed by the parents (Sample 1) all consisted of text and images. The length of the stories varied from 7 to 15 sentences (*M* = 9.35, SD = 2.37). An analysis of the SS indicated that 11 (65%, ranging from meeting 5 to 9 of the criteria) of the SS did not meet all of Gray's criteria, whilst 6 (35%) met all of the criteria. The SS which met all of the criteria (*n* = 6) resulted in a greater change in closeness-to-goal rating (*M* = 5.33, SD = 1.86) when compared to SS which did not meet all of Gray's criteria (*n* = 11, *M* = 3.73, SD = 3.00). However, this difference was not statistically significant, *M* = 1.61, 95% CI [−1.29, 4.50], *t*(15) = 1.18, *p* = .195, *d* = 0.64. As shown in Table 5, the CYP met most of Gray's criteria when developing their own SS (Sample 2). It is important to note that by writing the SS themselves, some criteria are automatically met (e.g., Two-step discovery, Gather information from the child and have a clear focus for the story; Nine makes it mine, SS should be tailored to meet the interests and individual needs of the child).

### Analysis of goals

Of the experimental goals identified by parents (Sample 1), 40% (*n* = 7) aimed to assist in transitions, novel situations or reduce anxiety (e.g., to get ready for school on time), 24% (*n* = 4) aimed to teach academic/functional skills (e.g., to attempt completing the English homework daily as independently as possible), 18% (*n* = 3) aimed to reduce inappropriate behaviours (e.g., not get upset if someone says no), and 18% (*n* = 3) aimed to improved appropriate social behaviours (e.g., communicating so others understand me). CYP (Sample 2) mostly (*n* = 3, 60%) developed goals to teach academic/functional skills (e.g., improve my attention span). There was also one SS to improve social behaviour (to make new friends) and one SS to prepare for an event (a trip to Gozo) (Table 6).

**Table 6:** SS goals developed by each sample.

SS category	Sample 1 (Parents)		Sample 2 (CYP)	
	Frequency	Percentage	Frequency	Percentage
Improve appropriate social behaviours	3	18	1	20
Reduce inappropriate behaviours	3	18	0	0
Teach academic/functional skills	4	24	3	60
Preparing for change/event; reduce anxiety	7	40	1	20

### Intervention evaluation

After the 2-week intervention, an evaluation was completed by parents from Sample 1. There was very strong agreement that digitally-mediated SS were both appropriate for CYP and that digital mediation was feasible (Table 7).

**Table 7:** Overview of evaluation questions for sample 1 (parents).

	Mean	SD	Min-Max
The digitally-mediated social story meets my approval	4.76	0.44	4–5
The digitally-mediated social story meets my child/adolescent's approval	4.47	0.80	2–5
Digitally-mediated social stories were appealing to my child/adolescent	4.47	0.72	3–5
My child/adolescent seems to have liked digitally-mediated social stories	4.53	0.62	3–5
My child/adolescent seems to have welcomed digitally-mediated social stories	4.71	0.59	3–5
Digitally-mediated social stories seem fitting for autistic CYP	4.41	0.80	3–5
Digitally-mediated social stories seem suitable for autistic CYP	4.35	0.79	3–5
Digitally-mediated social stories seem applicable for autistic CYP	4.35	0.79	3–5
Digitally-mediated social stories seem like a good match for autistic CYP	4.41	0.80	3–5
Digitally-mediated social stories seem implementable	4.41	0.51	4–5
Implementing digitally-mediated social stories seems possible	4.41	0.62	3–5
Digitally-mediated social stories seem doable	4.65	0.49	4–5
Digitally-mediated social stories seem easy to use	4.65	0.61	3–5

SD, standard deviation; Min, minimum rating; Max, maximum rating.

Finally, the CYP (Sample 2) were asked about their experiences of using the digitally-mediated SS. As shown in Table 8, there was very strong agreement with the statement that participation was a positive experience. Table 9 highlights there was full compliance with the protocol. It is important to note that some CYP needed reminders and support from parents, and this was the case for the two children (aged 7–9). The three young people (aged 14–16) reported that they did not require any additional support.

**Table 8:** Overview of evaluation questions for sample 2 (CYP).

	Mean	SD	Min-Max
I enjoyed participating in this study	4.20	0.45	4–5
I have benefited from participating in this study	4.20	0.45	4–5
I have met the goal which was identified at the beginning of the study	4.20	0.84	3–5
During the past week, I enjoyed using my smartphone or tablet	5.00	0.00	5–5

SD, standard deviation; Min, minimum rating; Max, maximum rating.

**Table 9:** Compliance for sample 2 (CYP).

	Frequency	Percentage
Did you read your story, on your smartphone or tablet, for at least once every day?		
Yes	5/5	100
Did you need to be reminded by an adult to read the story on the SOFA-app?		
Yes	2/5	40
No	3/5	60
Did you require support from an adult to complete the study?		
Yes	2/5	40
No	3/5	60

## Discussion

This study is the first study to investigate how a digitally-mediated SS intervention can be utilised in a naturalistic setting, by both parents of autistic CYP and autistic CYP themselves. Whilst none of the participants from Sample 1 of the study reached their goals completely (i.e., rated their closeness-to-goal as a 10), when compared to outcomes of the control story, the use of a digitally-mediated SS resulted in a statistically significant change in closeness-to-goal ratings for the experimental goal. This indicates that digitally-mediated SS intervention is an effective parent-mediated intervention (PMI), consistent with improvements observed when researchers and school teachers develop and deliver the intervention [(38, 39, 56); see also (14, 15, 23)]. Indeed, a larger effect size was observed in the present study for parents than has been reported for researchers and school teachers (64) which may relate to the high degree of parental involvement in the intervention. It is possible that intimate knowledge of children's profiles could be impacting positively the outcomes of the intervention. This finding supports further the involvement of parents in the CYP's support and treatment plans (5). Digital mediation may also provide support opportunities which are flexible and affordable; where parents can access training in their own time (65).

For the first time, this study also highlights the utility of digitally-mediated SS as a means of self-support and self-management for autistic CYP (Sample 2). Significant improvements were identified in closeness-to-goal ratings after 1 week of intervention. This improvement was also confirmed by parental reports. Previous studies focused mostly on self-management within clinical or school settings. Furthermore, reviews by Aljadeff-Abergel et al. (48) and Chia et al. (47) highlight the need for more research on self-management interventions in naturalistic and real-world settings. This study indicates that digitally-mediated SS intervention can support autistic CYP with identifying their own goal, develop and deliver their own stories, and monitor their progress towards their goals. Thus, this study also suggests that digitally-mediated SS could support self-mediated intervention in a naturalistic setting (i.e., outside a clinical setting). Whilst this was totally autonomous for autistic adolescents, autistic children did report needing support and reminders from parents to comply with the intervention protocol. With this proviso, the study suggests that the digital platform used to develop and deliver the story in the present study may support independence and self-determination (i.e., the ability to make choices and decisions about their own intervention goals) for autistic CYP.

The study also investigated elements of the intervention, as well as CYP's characteristics, which can impact the effectiveness of SS. In previous research, the audience's (autistic CYP) language skills (receptive/expressive) have been identified as a factor that impacts SS effectiveness positively (26, 27). The findings of the present study are consistent with this: whilst overall outcomes were positive across all participants, higher receptive/expressive language skills resulted in greater improvement in closeness-to-goal ratings. Systemizing was also found to be a CYP characteristic which influenced change in closeness-to-goal, with higher systemizing predicting greater improvement in closeness-to-goal outcomes. This provides some support for the suggestion that systemising plays an important role in the effectiveness of SS for autistic people. That is, SS provide rules and patterns which help to predict behaviour, and thus utilise a strength for input-operation-output relationships and a preference for explicit rule-based communication (29).

From the stories which were developed by the parents, only 35% met all of Gray's (18) criteria. This is however a higher percentage than what was reported by Smith, Toms, et al. (41) who carried out a similar study with school teachers. The question of the necessity for stories to meet all of Gray's criteria for them to be effective has been contentious (23, 66). This study indicates that there is no significant difference, in terms of change in closeness-to-goal outcomes, between the stories that met all of the criteria and those that met some of the criteria. However, the stories which met all of the criteria obtained a relatively higher change in closeness-to-goal ratings from baseline to outcome, when compared to those which did not (although not statistically significant). In addition, autistic CYP were largely able to develop stories consistent with Gray's criteria.

An element which was important for the effectiveness of SS was Gray's second criterion, which encourages authors to engage with the audience's perspective and gather information to inform the stories which are developed. The authors who reported having consulted with their children, and thus meeting Gray's second criterion, reported higher closeness-to-goal ratings than authors who did not. This finding emphasises the significance of Gray's notion of social humility in terms of the authors' recognition of their limits as a means of promoting empathy and respect towards the audience's perspective.

It is interesting to note that around one-fifth of both samples developed social stories to address the goal of improving social behaviour. When social stories are developed by practitioners, improving social behaviour is one of the most frequently identified goals [see (25)]. However, self-developed and self-delivered social stories have recently been found to be effective for autistic adults, who similarly, rarely chose a goal of improving social behaviour (52). One potential implication, amongst others, is that the autistic and broader autism communities have different intervention priorities from those of practitioners and professionals. Thus, self-development and self-delivery of the intervention in the present study, which is consistent with the goals of participatory autism research [e.g. (50)], can shed light on goals and supports which are relevant for the autistic community.

Finally, both Sample 1 and Sample 2 of the study can shed further light on the social validity of the digitally-mediated SS intervention. Parents from Sample 1 of the study, and autistic CYP from Sample 2 of the study, rated the acceptability and feasibility of the digitally-mediated SS intervention very positively. This confirms that digitally-mediated interventions are perceived as acceptable and useful in terms of procedure and outcomes by the autistic and broader autism communities.

## Limitations

There are a number of limitations to the present study. Sample 1 and Sample 2 both constituted small sample sizes, although they were consistent with the numbers recommended by power analyses. In both samples, the CYP's age range was broad (4–15 for Sample 1, and 7–16 for Sample 2). CYP in Sample 2 were all males. Parents, however, were mostly mothers, largely well-educated with high degrees of literacy (self-reported), as were the CYP in Sample 2 (parent-reported). The sample therefore may not be generalisable to parents and CYP with lower levels of literacy skills.

Another limitation of the study is that the diagnosis of the CYP in Sample 1 were reported by parents (as having received a formal diagnosis from clinicians by employing DSM-IV/V criteria; American Psychiatric Association, 2013), and the AQ-10 was used to confirm the diagnosis. However, given that the study was carried out remotely, the diagnosis could not be confirmed through other means. The diagnoses of Sample 2 were confirmed with an ADOS. However, the AQ10 scores did not differ between Sample 1 and Sample 2.

Furthermore, as a result of the remote modality of this study, it was not possible to ascertain the level of prompting parents provided the CYP to facilitate a desired outcome. Prompting, of the verbal and non-verbal kind, could have impacted to various degree the outcomes reported by the parents and also by the CYP. In future research designs measures of the quality and quantity of potential prompts can be included.

Another limitation is the manner in which progress towards the goal was measured; no objective measures were identified. Parents, as well as CYP, who participated in the study, were asked to monitor and measure their own progress towards their goals through an 11-point Likert scale. Such practice could have been prone to experiencing “demand characteristics”. As a result, participants could have overestimated the degree of change which was measured on the experimental goal which was selected. However, this does not seem to be the case as both positive and negative outcomes were reported in terms of closeness-to-goal measures. Although in Sample 2 of the study, the parents themselves acted as independent raters of their children's closeness-to-goal ratings, clearly parents are not fully independent from their CYP. Therefore, future research could explore conducting a “blind” evaluation, wherein evaluators are unaware of which goal serves as the experimental one and which acts as the control. Additionally, future studies could adopt a research design involving two groups of parents: one employing Social Stories (SS) for the experimental goal, while the other utilizes an alternative method to focus on their experimental goal.

For Sample 1, the parents were invited to consult with their CYP about the experimental goal, whilst the control goal was always identified solely by the parents. This could have impacted the relevance of the control goal for the CYP. In future, research can randomise which goal is to be experimental and which control. For Sample 2, the control goals were not included. Future research could include control goals in the research design.

Finally, the research design used for Sample 1 differed from that of Sample 2: i.e., the PMI study was carried out over 2 weeks, whilst the SMI was carried out over 1 week. The reason for this change was a result of the feedback obtained from parents when identifying CYP participants for Sample 2. Thus, direct feedback from participants impacted the research design. As a result, it was not possible to compare outcomes of a 2-week design with a 1-week design. However, in this study, the duration did not seem to affect the outcomes as significant and large effect sizes were identified for both the 2-week and the 1-week study. However, future research could investigate further if duration of the intervention could influence efficacy. Furthermore, the factors that influence autistic individual's engagement with an intervention or support tool, such as duration, could also be investigated further.

## Implications and conclusions

This exploratory study indicates that digital mediation can support parent-led and CYP-led SS support; specifically, that parents and autistic CYP may be supported in reaching their goals through a digitally-mediated SS intervention. Digital mediation of SS can support PMIs in terms of treatment fidelity, in a naturalistic setting, with little to no researcher input. Receptive and expressive language skills, together with systemizing, were identified as participant (CYP) characteristics which impacted intervention outcomes. On the other hand, the practice of parents consulting with the CYP at the development stage of the intervention was a characteristic of the intervention which impacted positively on outcomes. Furthermore, digitally mediated SS can be utilised by autistic CYP, who reported functional English reading and writing skills, as means of self-support. Digital mediation of SS can also support SMIs in terms of treatment fidelity, in a naturalistic setting, with little to no researcher input (though some parent support may be needed for younger children). Supporting the autistic and broader autism communities in pursuing their own goals may have great value and significance for the individuals concerned and for participatory research designs.

## Data Availability

The raw data supporting the conclusions of this article will be made available by the authors, without undue reservation.

## Footnotes

^1^ https://youtube.com/playlist?list=PL4OnsluvuH8Hg0Lb538N8dCfhcij8Ucla
